# Breast Cancer and Mental Health: Incidence and Influencing Factors—A Claims Data Analysis from Germany

**DOI:** 10.3390/cancers16213688

**Published:** 2024-10-31

**Authors:** Alexandra von Au, Dominik Dannehl, Tjeerd Maarten Hein Dijkstra, Raphael Gutsfeld, Anna Sophie Scholz, Kathrin Hassdenteufel, Markus Hahn, Sabine Hawighorst-Knapstein, Alexandra Isaksson, Ariane Chaudhuri, Armin Bauer, Markus Wallwiener, Diethelm Wallwiener, Sara Yvonne Brucker, Andreas Daniel Hartkopf, Stephanie Wallwiener

**Affiliations:** 1 Department of Gynecology and Obstetrics, Heidelberg University, 69120 Heidelberg, Germany; annasophie.scholz@med.uni-heidelberg.de (A.S.S.); kathrin.hassdenteufel@med.uni-heidelberg.de (K.H.); 2 Department of Women’s Health, Tübingen University, 72076 Tübingen, Germany; dominik.dannehl@med.uni-tuebingen.de (D.D.); tjeerd.dijkstra@med.uni-tuebingen.de (T.M.H.D.); gutsfeldr@gmail.com (R.G.); markus.hahn@med.uni-tuebingen.de (M.H.); armin.bauer@med.uni-tuebingen.de (A.B.); diethelm.wallwiener@med.uni-tuebingen.de (D.W.); sara.brucker@med.uni-tuebingen.de (S.Y.B.); andreas.hartkopf@med.uni-tuebingen.de (A.D.H.); 3 Institute for Translational Bioinformatics, University Hospital Tübingen, 72076 Tübingen, Germany; 4 AOK Baden-Wuerttemberg, 70188 Stuttgart, Germany; sabine.knapstein@bw.aok.de (S.H.-K.); dr.med.alexandra.isaksson@bw.aok.de (A.I.); dr.med.ariane.chaudhuri@bw.aok.de (A.C.); 5 Department of Gynecology, Halle University, 06120 Halle, Germany; markus.wallwiener@uk-halle.de; 6 Department of Obstetrics and Perinatal Medicine, Halle University, 06120 Halle, Germany; stephanie.wallwiener@uk-halle.de

**Keywords:** breast cancer, mental illness, depression, anxiety, risk factors, endocrine treatment, mastectomy

## Abstract

Background/Objectives: With breast cancer (BC) survival improving due to optimized therapy, enhancing quality of life has become increasingly important. Both diagnosis and treatment, with their potential side effects, pose risks to mental well-being. Our study aimed to analyze the incidence and potential risk factors for mental disorders in BC patients. Methods: This retrospective analysis used claims data from AOK Baden-Wuerttemberg, including 11,553 BC patients diagnosed via ICD code C50 between 2010 and 2020 and 31,944 age-matched controls. Patients with mental disorders in the 12 months prior to diagnosis were excluded. Mental disorders were categorized into eight groups based on ICD codes: anxiety, obsessive compulsive disorder, adjustment disorder, dissociative disorder, hypochondriac disorder, affective disorder, mania, and other neuroses. Results: Mental disorders were significantly more common in BC patients than in controls (64.2% vs. 38.1%, *p* < 0.01, OR 2.91, 95%CI [2.79, 3.04]). In particular, hypochondriac, anxiety, affective, and adjustment disorders occurred significantly more often in BC patients. No differences were found for mania, bipolar disease, other neuroses, obsessive compulsive-, or dissociative disorders. Furthermore, endocrine therapy was associated with psychological comorbidities (OR 1.69, *p* < 0.001, 95%CI [1.53, 1.86]), while primarily metastasized patients (stage C) had a lower risk than adjuvant patients in stage A (OR 0.55, *p* < 0.0001, 95%CI [0.49, 0.61]). Regarding surgical treatment, mastectomy patients showed lower rates of mental illnesses (61.2%) than those with breast-conserving treatment (71.6%), or especially breast reconstruction (78.4%, *p* < 0.01). Breast reconstruction was also associated with more hypochondriac (*p* < 0.01) and adjustment disorders (*p* < 0.01). Conclusions: So, BC patients experience significantly more mental disorders than controls, particularly when treated with endocrine therapy and breast reconstructive surgery.

## Simple Summary

This study investigates how frequently breast cancer patients develop mental disorders compared to a control group without breast cancer. We analyzed data from over 11,000 breast cancer patients and nearly 32,000 healthy controls, finding that breast cancer patients were significantly more likely to suffer from mental disorders. Anxiety disorders, hypochondria, adjustment disorders, and depression were particularly common. This study also examined factors such as the type of therapy: endocrine therapy and breast reconstruction were strongly associated with psychological distress, and simple-mastectomy patients showed the lowest rates of mental illness. This work is of particular importance because it highlights how significantly the mental health of breast cancer patients can deteriorate due to their diagnosis and treatment. Thus, psychological counseling should be part of every treatment for breast cancer, especially when endocrine therapy is recommended. The practice of recommending breast reconstruction after mastectomy should be reconsidered, as it could have a negative effect on mental health.

## 1. Introduction

In 2020, female breast cancer (BC) was the most commonly diagnosed cancer worldwide, at 11.7% [1], and should be considered a global health concern, especially due to the rising incidence rates anticipated in regions of the world that are currently undergoing economic transformation [1,2]. With BC therapy continuously being optimized and, hence, survival improving over the last few decades [3], reducing comorbidities and enhancing quality of life have become increasingly relevant aspects of therapy [4]. Both the cancer diagnosis itself and the treatment, with its potential side effects, are often intensely distressing and pose a risk to mental well-being [5,6,7,8]. However, the prevalence rates of mental disorders in cancer patients vary widely in the literature, mainly as a result of inconsistency in the use of measurement tools and differences in methodological approaches [9,10,11,12,13]. So, for diseases within the depressive spectrum alone, a review by Caruso et al. reported figures between 5% and 60% that were measured in past analyses [14]. In two more recent meta-analyses focusing on BC patients, the risk of depression was measured at 32.2%, while the risk of anxiety was measured at 41.9%, respectively [15,16]. In principle, it must therefore be assumed that mental illness constitutes a relevant comorbidity in cancer patients in general, and especially in BC patients. Such psychological distress is also associated with negative effects on health outcomes and may impact the course of the cancer disease and compliance [17]. The results of a meta-analysis demonstrated that depression functions as a negative predictive factor for adherence to endocrine treatment [18]. Another review from 2020 suggests that depression and anxiety disorders in BC patients may even be associated with higher mortality [19]. While studies on depression and anxiety disorders in cancer patients are increasing, there is still a lack of valid data regarding the occurrence of other mental illnesses in cancer patients. Thus, the influence of the psyche on the treatment and prognosis of BC has apparently not been fully clarified yet.

Similarly, the question of why some breast cancer patients develop psychological comorbidities while others do not remains unresolved. To date, several risk factors for mental diseases in the context of cancer diagnosis and treatment have been discussed. Evidence suggests that factors such as age at diagnosis and disease stage may play a role in the development of mental disorders following a cancer diagnosis: Ref. [12]. Some studies indicate that an older age is associated with more psychological comorbidities in breast cancer patients [20], while others link younger age to a higher incidence of mental health issues [21,22,23]. Regarding disease stage, the current literature supports the notion that a higher disease stage, especially metastatic disease, is associated with increased risk [11].

The evidence regarding the influence of a familial predisposition to cancer on the development of mental disorders is heterogeneous. The potential negative impact of an existing gene mutation is currently under discussion [24,25]. Not only patient- and tumor-specific factors, but also various therapies for cancer patients appear to have an impact on mental health. Chemotherapy or radiation therapy are discussed as potential promotors of mental comorbidities, as they cause significant disruptions in the lives of cancer patients due to pronounced systemic side effects, such as menopausal symptoms, cognitive dysfunction, and fatigue [26,27]. For example, Costanzo et al. stated that women who received chemotherapy reported greater anxiety than those who received only radiation therapy [23]. This finding seems logical, as there is a strong association between fatigue and mental disorders in cancer patients. Additionally, other researchers have noted that chemotherapy patients tend to suffer more from chronic fatigue than those undergoing radiation therapy, which is often associated with more acute fatigue effects [28,29]. Another important aspect regarding chemotherapy is the early and comprehensive education of the patient about treatment recommendations, ideally involving her in the decision-making process. This approach has been associated with reduced distress [30]. Furthermore, in the context of shared decision-making, it is essential to consider the ongoing discussion about the psychological impact of different surgical treatment options. Particularly the removal of one or both breasts may be associated with increased depressive symptoms and body image issues [31]. Not only surgical techniques, but also endocrine treatments exert an influence on well-being. Due to their multiple side effects, particularly menopausal symptoms, these treatments are linked to a decrease in quality of life [32,33]. A recent study from China even suggested that the side effects of endocrine therapy are significantly associated with anxiety and depression [34]; however, this was a relatively small study. Thus, valid data on the impact of the aforementioned factors are currently lacking but are urgently needed to support recommendations for more intensive care based on specific patient characteristics, tumor characteristics, and treatment modalities.

So, the primary objective of this study was to analyze the incidence of different mental disorders in BC patients compared to the general population. As a secondary aim, we sought to clarify whether the aforementioned potential risk factors contribute to the development of mental disorders in BC patients. Ultimately, the goal of our study is to facilitate a more individualized, patient-centered approach to care in the future.

## 2. Materials and Methods

**Figure 1:**
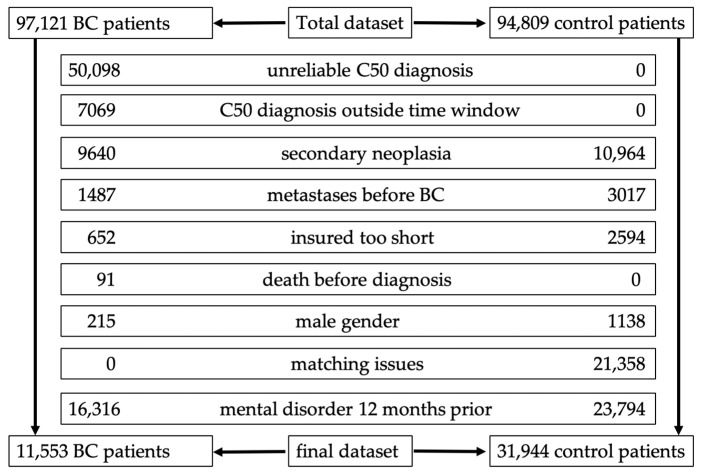
Flow chart regarding exclusion criteria.

The anonymized dataset provided by AOK Baden-Wuerttemberg consisted of 97,121 patients (95,499 women and 1622 men) who received a BC diagnosis (International Statistical Classification of Diseases and Related Health Problems (ICD) 10 code “C50”) and 94,849 age-matched control patients (also 93,253 women and 1596 men) without a BC diagnosis between January 2010 and December 2020 (observation period): Ref. [35]. For analysis in our BC group (BCG), we initially had to exclude 69,252 patients, either due to unreliable C50 diagnosis (no in-patient treatment for invasive BC between 1 July 2010 and 31 December 2019, the analysis period), C50 diagnosis outside the time window of the analysis period, encoding of secondary neoplasia (before or after the first diagnosis of invasive BC, except nonmelanoma skin cancer, ICD10 code C44), occurrence of distant metastases at least 6 months prior to the first encoding of C50, a too-short period of insurance coverage (overall insurance duration less than 40% of the observation period), death before diagnosis, or male gender; see Figure 1.

From our control group (CG) we also had to exclude 39,071 patients owing to missing data concerning the insurance period or a too-short period of insurance coverage, the development of a neoplastic disease (except for nonmelanoma skin cancer, ICD10 code 44), male gender, and due to matching. As previously described, matching was performed to pair each BC patient with two unique patients in the control group (1:2 ratio). Matching was implemented using the R package optmatch. Age at first diagnosis was selected as the main matching criterion. As the second matching criterion, a “no exclusion before diagnosis” constraint was applied. For a more detailed description of our matching process, we refer to our prior publication: Ref. [35]. As also described in our previous publication, we differentiated three distinct breast cancer stages: 1. Stage A, early BC without pathological axillary lymph node involvement (encoding of C50 without encoding of C77.3); 2. Stage B, early BC with pathological axillary lymph node involvement (encoding of C77.3 within 6 months of BC diagnosis date); and 3. Stage C, primary distant metastatic BC [encoding for distant metastatic disease by C77–C79, except C77.3 (axillary lymph node involvement) and C77.9 (lymph node involvement, not otherwise specified) within 6 months of BC diagnosis date] [35]. The BC histologic subtypes were reconstructed from the medication the patient received according to the Anatomic Therapeutic Chemical codes (ATC) recorded [36]. If our BC patients received the corresponding medication at least once in the observation period after the first diagnosis of C50, they were defined as either hormone receptor (HR)- or HER2-positive; otherwise, they were assumed to be HR- or Her2-negative.

For this analysis regarding mental disorders in BC patients, we excluded an additional 16,316 BC patients in whom a mental disease was diagnosed 12 months (4 quarters) before their BC diagnosis and 23,794 control patients who received a mental illness diagnosis 12 months prior to the BC diagnosis of the matched BC patient; see Figure 1. For our analysis, the following ICD10 codes were used to categorize mental diseases: F32–34 and F38 were summarized as affective disorders, F40 and F41 for anxiety disorders, F42 for obsessive compulsive disorders, F45 for somatic symptom disorders and hypochondriac disorders, F44 for dissociative disorders, F30 and F31 for mania and bipolar disorders, F43 for adjustment disorders, and F48 for other neurotic disorders. In some of our analyses F40–42, F44–45, and F48 were regarded together as neurotic disorders. For patient characteristics, the following ICD10 codes/EBM codes were used: diabetes mellitus ICD10 E10–E13, obesity ICD10 E66, and hereditary BC/familial BC predisposition ICD10 Z40+Z80, EBM 11440/11518/11601/11230/11233.

### Statistics

Data processing and statistical analysis were performed using R version 4.3.3 and RStudio (version 2024.04.0+735, Posit PBC, Boston, MA, USA) with the tidyverse packages dplyr 1.1.4, readr 2.1.5, forcats 1.0.0, stringr 1.5.1, ggplot2 3.5.1, tibble 3.2.1, lubridate 1.9.3, tidyr 1.3.1, and purrr 1.0.2. For generating tables, we used the packages janitor 2.2.0, gt 0.11.0, and gtsummary 2.0.0. Kaplan–Meier curves were generated using the packages survival 3.7-0 and ggsurvfit 1.1.0 to estimate the time to disease together with the associated 95% confidence intervals (CIs). We used chi-squared tests to compare the BC group and the control group. Given the large group sizes, *p*-values were often extremely small, thus we considered the effect sizes to gauge the significance. In detail, we used Cohen’s D and W measures to quantify effect size calculated with the package effect size 0.8.9. The incidence of mental diseases was registered using the ICD10 codes. To analyze factors affecting the occurrence of a mental disorder after BC diagnosis, we applied a logistic regression model. We considered factors significant when they had a *p*-value < 0.001.

## 3. Results

### 3.1. Patient Characteristics

After applying the aforementioned inclusion and exclusion criteria, a total of 43,497 patients (11,553 BC patients and 31,944 control patients) were included in the analysis; see Figure 1. The mean age of our BC patients was 66.1 years versus 65.0 years in the control group (CG). Table 1 displays the baseline patient characteristics that were reconstructed using claims data as described in the Materials and Methods section as well as in our previous publication [35].

Regarding the general characteristics in both groups, the majority of patients lived in the suburbs (BCG 41.7%; 4813/11,553 versus CG 42.1%; 13,399/31,944), and about half of each group participated in GP-centered care programs (BCG 52.9%; 6112/11,553 versus CG 49.8%; 15,922/31,944). About one-fourth of each group’s patients presented with diabetes mellitus (BCG 28.2%; 3256/11,553 versus CG 25.2%, 8057/31,944), and one-third presented with obesity (BCG 32.8%; 3788/11,553 versus CG 30.9%; 9884/31,944). At 19% (2194/11,553), significantly more patients in the BCG had a familial BC predisposition as compared to 6.1% (2074/31,944) of the controls (*p* < 0.05; Cohens D 0.186 small effect). Regarding the BC patients in this analysis, the most common tumor subtype was HR+/HER2− (7996/11,553; 69.2%), followed by HR−/HER2− (2528/11,553; 21.9%), HR+/HER2+ (709/11,553; 6.1%), and HR−/HER2+ (320/11,553; 2.8%). Furthermore, the majority of our BC patients (7775/11,553; 67.3%) were assigned to stage A, 17.2% to stage B (1990/11,553), and 15.5% to stage C (1788/11,553). Furthermore, 75.3% (8705/11,553) of BC patients received endocrine treatment, 81.1% (9373/11,553) breast surgery, and 62.4% (7214/11,553) radiation therapy, and 34.7% (4008/11,553) were treated with chemotherapy.

### 3.2. Mental Illnesses in BC Patients

Concerning our primary objective, we found that at 64.2% (7418/11,553) our BC patients developed a mental disorder significantly more often than our control patients (38.1% (12,176/31,944); *p* < 0.01; OR 2.91, 95%CI [2.79, 3.04]). Regarding each psychological illness separately, we also found significantly higher rates among BC patients for anxiety (BCG 19.3%; 2229/11,553 versus CG 7.6%; 2441/31,944, *p* < 0.01 OR 2.89, [2.72, 3.07]), somatic symptom disorders and hypochondriac disorders (BCG 35.1%; 4055/11,553 versus CG 17.6%; 5617/31,944, *p* < 0.01, OR 2.54, [2.42, 2.66]), affective disorders (BCG 34%; 3927/11,553 versus CG 18.9%; 6025/31,944, *p* < 0.01, OR 2.22, [2.11, 2.32]), and adjustment disorders (BCG 27.2%; 3137/11,553 versus CG 12.2%; 3894/31,944, *p* < 0.01, OR 2.69, [2.55, 2.83]); see Table 2.

**Figure 2:**
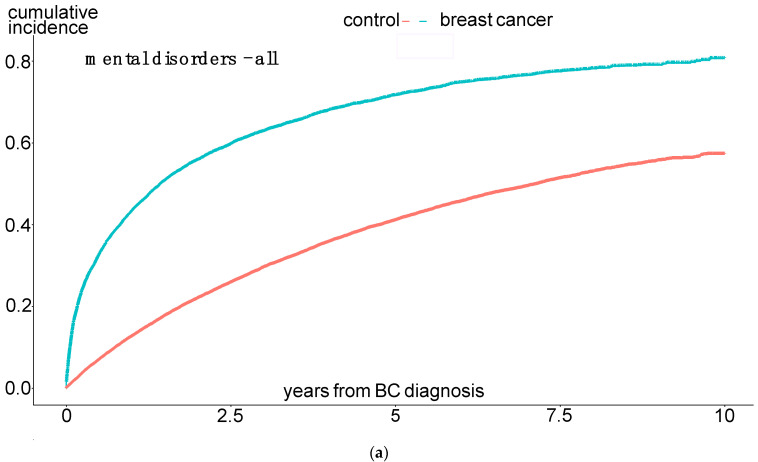

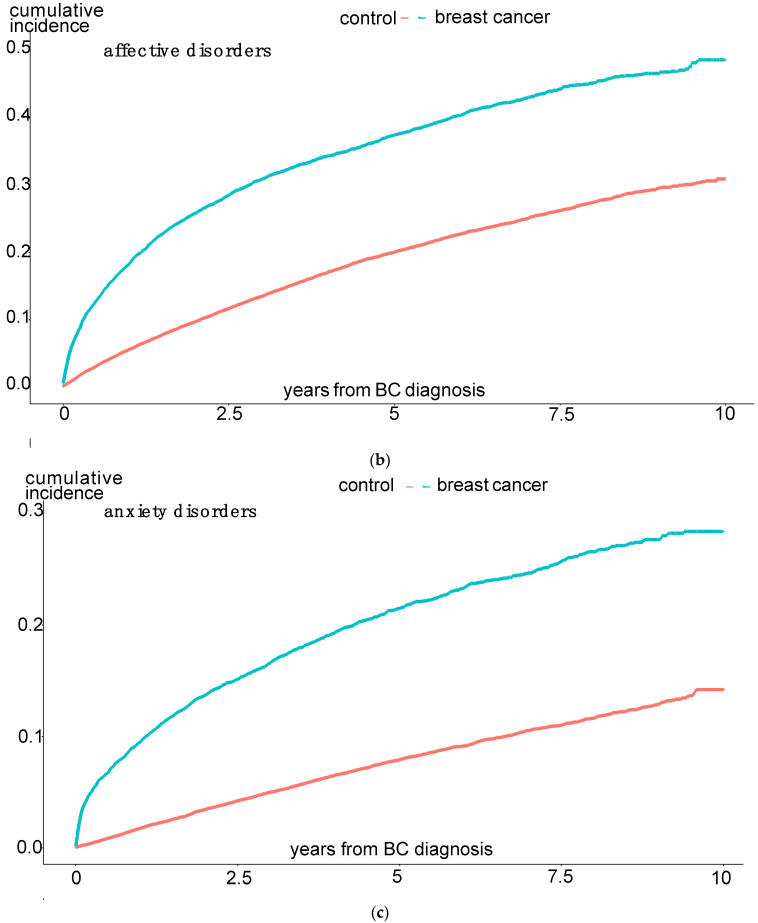

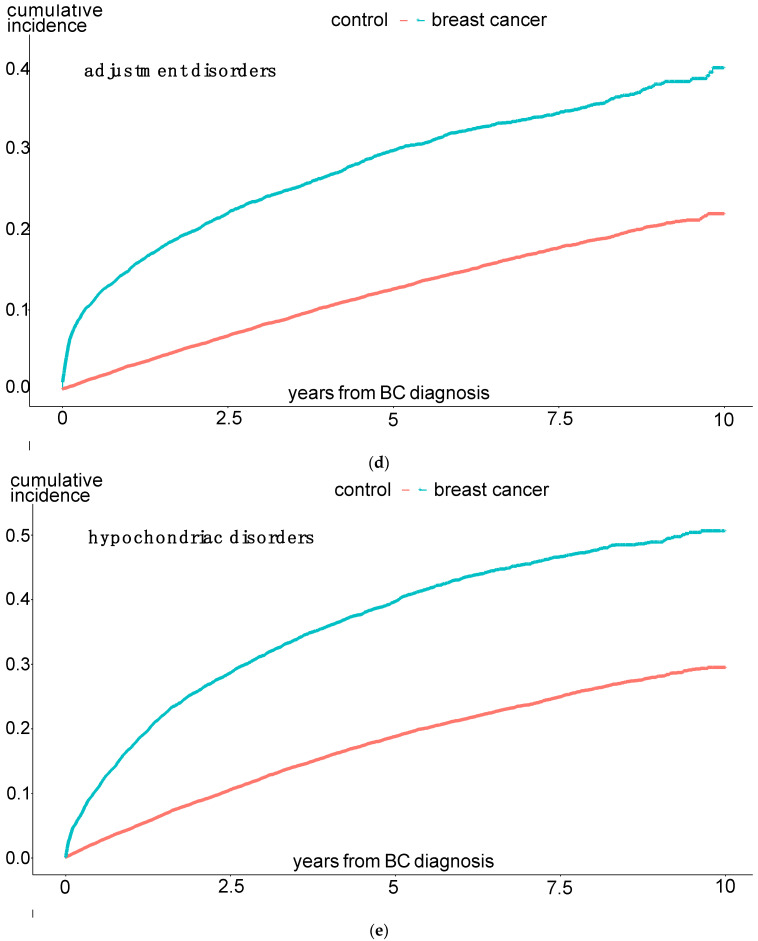
Differences in the occurrence of various mental disorders between BC patients and the control group; (**a**) mental disorders, (**b**) affective disorders, (**c**) anxiety disorders, (**d**) adjustment disorders, and (**e**) hypochondriac disorders. Time-to-event analysis (presentation of cumulative incidences in % (*y*-axis) for the occurrence of the respective event (mental disorder, adjustment disorder, etc.) over a 10-year observation period (*x*-axis).

The incidence rates of mania and bipolar disorders, obsessive compulsive disorders, dissociative disorders, and “other neuroses” did not differ between the groups. Furthermore, Kaplan–Maier curves were generated to analyze the cumulative incidences of mental diseases over a 10-year observation period. Here, the same significant differences between the BC and control patients were observed: the total number of mental disorders, especially affective disorders, anxiety disorders, hypochondriac disorders, and adjustment disorders, were significantly more common among BC patients (see Figure 2a–e).

The results in Figure 2 show that these patients develop the mental disorder particularly within the first year after the BC diagnosis, i.e., while still undergoing treatment. To further investigate the extent to which the therapy directly influences the occurrence of a psychological comorbidity, we examined the experimental group in more detail.

### 3.3. Risk Factors for Mental Illnesses

Furthermore, a logistic regression model was applied to assess the occurrence of mental illness considering the following factors: chemotherapy, radiation therapy, mastectomy, endocrine therapy, age at first diagnosis, stage of BC, and family history of BC predisposition, see Table 3. An OR >1.68 or <0.59 with a *p*-value < 0.001 was considered a significant difference.

The analysis of the various treatments revealed that endocrine therapy, with an OR of 1.69 (*p* < 0.0001, small effect size, 95% CI [1.53, 1.86]), was significantly associated with an increased occurrence of mental illness. For the other therapies examined, only a trend toward more mental illness was observed (chemotherapy OR 1.33, no effect, [1.21, 1.47]; radiation therapy OR 1.53, no effect, [1.39, 1.68]; and mastectomy OR 1.31, no effect, [1.19, 1.45]). Furthermore, primarily metastasized patients (stage C) had a significantly lower risk of being diagnosed with a mental illness compared to those with early breast cancer without lymph node involvement (stage A) (OR 0.55, *p* < 0.0001, small effect, [0.49, 0.61]). In this model, no significant difference was found for stage B compared to stage A (OR 0.87, *p* = 0.022, no effect, [0.78, 0.98]).

Regarding age at first diagnosis, we observed a trend toward more mental illness in younger patients, but, again, the effect size was not significant (OR 0.63, *p* < 0.001, no effect, [0.60, 0.66]). For patients with a family history of BC predisposition, there was also only a trend toward more mental illness (OR 1.24, *p* < 0.001, no effect, [1.11, 1.40]).

Since the initial model did not account for the fact that patients could have undergone breast-conserving surgery alone, as well as reconstructive breast surgery after mastectomy, this was examined in more detail in a subsequent analysis. Here, we conducted a further analysis and distinguished the surgical groups more precisely.

The BC patients were now divided into three groups based on their surgical therapy. The first group, “BCT”, included patients who received breast-conserving therapy with or without reconstructive surgery—essentially, all patients who never completely lost their breast. The second group, “Mastectomy,” included patients in whom mastectomy without reconstruction was performed (including primary mastectomy patients and patients with initial BCT followed by secondary mastectomy). The third group, “Reconstruction,” included all patients with primary or secondary mastectomy followed by breast reconstruction. Patients who, according to the coding, only received “reconstructive surgery” were excluded from our analysis, as they could not be clearly assigned to any of these three groups, and this does not represent a standard surgical therapy for an oncological breast procedure.

Thus, this analysis was conducted on a cohort of 9365 BC patients.

This analysis showed that patients in the mastectomy group did not have higher rates of mental disorders (see Table 4). On the contrary, a significantly lower proportion of patients in the mastectomy group (61.2%) suffered from a mental disorder compared to 71.6% in the BCT group, and 78.4% in the reconstruction group. Furthermore, 47.6% of patients in the reconstruction group showed significantly more hypochondriac disorders than patients in whom mastectomy was performed (30.9%). A significantly different result was also found for the development of adjustment disorders: they were also most common in the reconstruction group (43.1%) and least common in the mastectomy group (23.7%). Overall, the reconstruction group also showed more “other neuroses” and “affective disorders” than the mastectomy group in the post hoc analyses. No differences were found between the BCT and reconstruction groups in terms of the occurrence of a mental disorder. Regarding mania, dissociative disorder, and obsessive compulsive disorder, no significant differences were found between the three groups based on surgical therapy. In conclusion, our initial hypothesis that women who undergo mastectomy without subsequent reconstruction suffer more and therefore potentially develop more psychological comorbidities was not confirmed.

## 4. Discussion

With 64.2% of patients affected in our BC cohort, we observed significantly more mental disorders during the observational period compared to 38.1% in the healthy control patients; in particular, anxiety disorders, adjustment disorders, hypochondriac disorders, and affective disorders were more common. Thus, overall, we can confirm our previously stated main hypothesis that BC patients suffer from mental disorders more frequently. Considering the existing literature, the reported prevalence of depression and anxiety disorders among BC patients varies around approximately 30%. In a 2018 study by Tsaras et al., symptoms of depression or anxiety disorder were diagnosed in nearly 38% and 32%, respectively, of the included patients [11]. Similar results were seen in a study from Germany, showing a 5-year incidence of depression and/or anxiety disorder of 35.1% of BC patients treated in gynecological private practices [12]. Moreover, the analysis of our control group indicates that 38.1% of the women were affected by a mental illness, a figure consistent with representative data for Germany. A national survey from 2014 demonstrated that approximately one-third of adult women in Germany suffer from a mental disorder [37]. Thus, our results in this analysis both regarding the BC group and the control group are in line with the literature. In another analysis by Burgess et al., nearly 50% of BC patients in their first year after diagnosis suffered from depression and/or anxiety disorder [38]. As stated above, in our analysis, too, BC patients developed a mental disorder particularly within the first year after BC diagnosis. Such an effect was similarly observed in another claims data analysis from Korea [39] as well as in a systematic review including 17 original articles on the topic [40]. One possible explanation could be that the life-threatening diagnosis of BC represents a potential trauma with a corresponding risk of an acute or post-traumatic stress reaction and an adjustment disorder [41]. Already, in 2002, Amir et al. reported that the diagnosis of post-traumatic stress disorder is common among long-term BC survivors [42]. As BC patients who are diagnosed with a cancer-related acute stress disease are at heightened risk of a second mental disorder diagnosis [41], it is essential to provide these affected women with medical support at an early stage to prevent potential long-term mental health consequences.

Regarding the potential influencing factors, our most interesting finding was that patients who received endocrine treatment suffered from mental distress significantly more often (OR 1.69). The most commonly prescribed anti-endocrine therapies are the selective estrogen-receptor modulator (SERM) tamoxifen in premenopausal women and aromatase-inhibitors (AI) such as the third-generation agents anastrozole, letrozole, and exemestane in postmenopausal women: both therapies affect estrogen levels and the estrogen pathway in women, though in different ways [43]. Estrogen has a significant impact on mood and mental state, e.g., low estrogen levels in women are linked to premenstrual syndrome as well as postpartum and postmenopausal depression [44]. Thus, based on our finding that endocrine treatment is associated with higher rates of mental disorders, we support the hypothesis that it leads to psychological side effects in women. Considering these results, it is crucially important to educate BC patients about possible psychological side effects before starting the treatment as well. Currently, in Germany, the S3 guideline recommends an initial screening for psychological preconditions at the time of diagnosis during in-patient treatment, but there is no recommendation for routine follow-up or comprehensive psycho-oncological support for patients throughout the follow-up period [45]. However, due to the high number of affected BC patients, widespread psycho-oncological co-management is mandatory. This is particularly true for patients who receive endocrine treatment only instead of chemotherapy, as they are often not referred for regular psycho-oncological care. So ideally, follow-up psycho-oncological support should be implemented within the primary care framework at BC centers. Despite the common assumption that chemotherapy and radiation therapy represent a significant burden for BC patients, like other studies before, our analysis did not find an association with mental disorders for these treatment-associated factors [46]. Furthermore, being in stage C (primary distant metastatic BC) was associated with significantly lower numbers of mental disorders in our study. Assessing the clinical stage of disease in BC patients is a crucial step in determining both treatment strategy and prognosis [47]. A higher stage usually comes with a poorer prognosis and, with respect to the current literature [48], we initially assumed that higher disease stages would be associated with more psychological comorbidities. Possible explanations for our contradictory result could be that patients in advanced stages of cancer may prioritize physical survival and immediate medical concerns over mental health issues, thus the focus on survival could diminish the impact or at least the reporting of psychological distress. Furthermore, patients with more advanced disease might receive more comprehensive palliative care, including psychological support, which could help mitigate feelings of anxiety and depression. The awareness and acknowledgment of their prognosis might also lead to increased support from friends and family, contributing to better psychological outcomes.

Regarding surgical treatment, we found a significant correlation between reconstructive breast surgery and the occurrence of mental disorders, in general, and of both adjustment disorders and hypochondriac disorders in particular. Patients treated by mastectomy, on the contrary, showed the lowest rates of mental illness (61.2%); these patients were significantly less affected than those in the BCT group (71.6%) and in the breast reconstruction group (78.4%). Regarding the current literature, there are mixed reports on the impact of mastectomy on BC patients [49,50,51,52]. In a current meta-analysis by Mishra and colleagues, the authors stated that mastectomy has a strong negative impact on women’s appearance and psychology [31]. However, when examining the literature in more detail, it becomes clear that some studies report contradictory results. For example, a study from Italy comparing mastectomy patients with and without breast reconstruction to healthy women found no difference between mastectomy patients with versus without reconstruction in terms of the occurrence of anxiety disorders, quality of life, and social adaptation [53]. Additionally, many women regret undergoing breast reconstruction over time. A study from Australia on post-decision regret after breast reconstruction found that 27.6% of women experienced mild regret, while 19.5% experienced moderate to severe regret regarding their decision for breast reconstruction. In this study, decision regret was associated with depression, anxiety, low satisfaction with preparatory information, and stress [54]. Another prospective longitudinal survey study, which compared the psychological outcomes of mastectomy patients with those who underwent delayed breast reconstruction, showed that women with delayed breast reconstruction had significantly higher levels of total distress, obsessiveness, and cancer-related distress than those with mastectomy alone. Furthermore, in that study, no differences in quality of life between the two groups could be seen at any time point [55]. Nissen et al. also conducted a prospective study regarding quality of life, which revealed that women with mastectomy and following reconstruction showed greater mood disturbances and poorer well-being after baseline and still 18 months after surgery [52]. These factors highlight the complexity of psychological responses to different types of BC surgery, necessitating tailored mental health interventions across the disease spectrum. In most BC centers in Germany, women diagnosed with BC are already advised on the pros and cons of mastectomy versus breast-conserving therapy in the context of participatory decision-making. If there is no indication for mastectomy, BCT with subsequent radiation is usually offered as the standard option, and mastectomy alone is considered an “equivalent alternative”, with or without subsequent reconstruction. Some have argued that psychological stress potentially develops after breast amputation, and the German S3 guideline even speaks of a potential disadvantage if immediate reconstruction cannot follow a mastectomy [45]. However, from our analysis on over 9000 BC patients and also our current literature research, this argument cannot be supported. In the comparison of mastectomy, BCT, and breast reconstruction in the present study, breast-conserving and especially reconstructive therapy were associated with increased psychological distress, while a mastectomy was not. These results are challenging the current guidelines. We suggest that in the future, counseling should not only be more open to a woman’s desire for mastectomy without reconstruction but even outline that breast reconstruction is associated with a higher risk of mental health problems. Furthermore, patients undergoing reconstructive surgery should at least receive more intensive follow-up and possibly psychological support.

As we already discussed in our prior publication [35], retrospective claims data analyses are valuable tools in healthcare research, reflecting actual clinical practice in a broad population and offering insights into real-world treatment patterns and outcomes. The most important advantages of retrospective claims data analyses are the large sample sizes, like the one in our present investigation, making it a robust dataset for statistical analyses. However, claims data are primarily collected for billing purposes, and not research. Thus, the data provided on tumor stage, patient characteristics, or social determinants of health are not exact. Therefore, the issue of incomplete data poses a limitation on data analysis. In this analysis, for example, we had to exclude 50,098 patients due to an unreliable C50 diagnosis from the original dataset. Another challenge in claims data analysis in general and also for our study is establishing correct causal relationships. To ensure that the mental illness is indeed a consequence of BC diagnosis or treatment, we selected a cohort in which no psychological comorbidities had been diagnosed within the 12 months preceding the initial BC diagnosis. This approach allowed us to establish a clear temporal sequence of diagnostic events, albeit at the expense of cohort size, as we were required to exclude an additional 16,316 BC patients.

## 5. Conclusions

Our analyses indicate that patients with BC are at a significantly increased risk of developing a psychological disorder. In particular, adjuvant patients, those receiving endocrine treatment, and those treated with reconstructive breast surgery seem to be at higher risk. Thus, it is particularly important to educate BC patients also about possible psychological side effects before starting the treatment. Furthermore, reconstructive surgery should be openly discussed with the patients, as it is associated with more, instead of less, psychological distress. Our results challenge the current practice to generally recommend reconstructive surgery following mastectomy.

## Figures and Tables

**Table 1:** Basic characteristics of BC patients versus control group patients.

Characteristics	BC Patients	Control Group	*p*-Value	Effect Size Cohens W/ **** Cohens D/***** r
Number/N=	11,553 (26.6%)	31,944 (73.4%)		
Mean age	66.1 (SD 14.7)	65.0 (SD 14.5)	<0.05	0.079 **** no effect
Stage of BC at first diagnosis			-	
stage A *	7775 (67.3%)	-		
stage B **	1990 (17.2%)	-		
stage C ***	1788 (15.5%)	-		
Estimated biologic subtype			-	
HR−/HER2−	2528 (21.9%)	-		
HR−/HER2+	320 (2.8%)	-		
HR+/HER2−	7996 (69.2%)	-		
HR+/HER2+	709 (6.1%)	-		
Endocrine treatment			-	
no	2848 (24.7%)	-		
yes	8705 (75.3%)			
Breast surgery			-	
no	2180 (18.9%)	-		
yes	9373 (81.1%)	-		
Radiation therapy			-	
no	4339 (37.6%)	-		
yes	7214 (62.4%)			
Chemotherapy			-	
no	7545 (65.3%)	-		
yes	4008 (34.7%)	-		
Living area (urban density level)			<0.05	0.012 ***** no effect
rural	2941 (25.5%)	8364 (26.3%)		
suburban	4813 (41.7%)	13,399 (42.1%)		
urban	3790 (32.8%)	10,036 (31.6%)		
GP-centered care program			<0.05	0.027 no effect
no	5441 (47.1%)	16,022 (50.2%)		
yes	6112 (52.9%)	15,922 (49.8%)		
Diabetes mellitus			<0.05	0.030 no effect
no	8297 (71.8%)	23,887 (74.8%)		
yes	3256 (28.2%)	8057 (25.2%)		
Obesity			<0.05	0.018 no effect
no	7765 (67.2%)	22,060 (69.1%)		
yes	3788 (32.8%)	9884 (30.9%)		
Familial BC predisposition			<0.05	0.186 small
no	9359 (81%)	29,870 (93.5%)		
yes	2194 (19%)	2074 (6.5%)		

* Stage A (early breast cancer without pathologic axillary lymph node involvement, ** stage B early breast cancer with pathologic axillary lymph node involvement, *** stage C primary metastatic breast cancer; **** effect size “Cohens D”; ***** effect size “r”; GP = general practitioner; ICD10 codes/EBM codes: diabetes mellitus ICD10 E10-E13, obesity ICD10 E66, familial BC predisposition: ICD10 Z40+Z80, EBM 11440/11518/11601/11230/11233).

**Table 2:** Differences in the occurrence of various mental disorders between BC patients and control patients; specification of the incidence in percentage; specification of the risk in form of the OR. Significant differences are printed in bold letters.

	BC Group N = 11,553	Control Group N = 31,944	OR	95% CI	Cohens W Effect Size	*p*-Value
Mental disorder (total)	**7418 (64.2%)**	**12,176 (38.1%)**	**2.91**	**[2.79, 3.04]**	**0.232 small**	**<0.01**
Anxiety disorder	**2229 (19.3%)**	**2441 (7.6%)**	**2.89**	**[2.72, 3.07]**	**0.166 small**	**<0.01**
Obsessive–compulsive disorder	26 (0.2%)	60 (0.2%)	1.20	[0.76, 1.90]	0.004	0.52
Dissociative disorder	64 (0.6%)	148 (0.5%)	1.20	[0.89, 1.61]	0.006	0.26
Hypochondriac disorder	**4055 (35.1%)**	**5617 (17.6%)**	**2.54**	**[2.42, 2.66]**	**0.186 small**	**<0.01**
Other neuroses	1160 (10%)	1806 (5.7%)	1.86	[1.73, 2.01]	0.077	<0.01
Affective disorder	**3927 (34%)**	**6025 (18.9%)**	**2.22**	**[2.11, 2.32]**	**0.159 small**	**<0.01**
Mania and bipolar disorder	37 (0.3%)	88 (0.3%)	1.16	[0.79, 1.71]	0.004	0.50
Adjustment disorder	**3137 (27.2%)**	**3894 (12.2%)**	**2.69**	**[2.55, 2.83]**	**0.180 small**	**<0.01**

**Table 3:** Log. regression model considering the following factors: chemotherapy, radiation therapy, endocrine therapy, mastectomy, age at first diagnosis, stage of breast cancer, and familial breast cancer predisposition.

Factors	Log(OR)	SE	OR	95%CI	*p*-Value
Chemotherapy	0.29 **	0.051	1.33	[1.21, 1.47]	<0.001
Radiation therapy	0.42 **	0.048	1.53	[1.39, 1.68]	<0.001
**Endocrine therapy**	0.52 **	**0.049**	**1.69**	**[1.53, 1.86** **]**	**<0.001**
Mastectomy	0.27 **	0.050	1.31	[1.19, 1.45]	<0.001
Age at first diagnosis	−0.47 **	0.025	0.63	[0.60, 0.66]	<0.001
Stage of BC					
1 versus 2	−0.14 *	0.060	0.87	[0.78, 0.98]	0.022
**1 versus 3**	−0.60 **	**0.058**	**0.55**	**[0.49, 0.61** **]**	**<0.001**
Familial BC predisposition	0.22 **	0.059	1.24	[1.11, 1.40]	<0.001

Dependent variable: mental disease after BC diagnosis, R2 = 0.094 (low); * *p* < 0.05, ** *p* < 0.001; N = 11,553, event N = 7418. OR = odds ratio, SE = standard error, CI = confidence interval. OR > 1.68 or <0.59 with a *p*-value < 0.001 was considered a significant difference, printed in bold letters.

**Table 4:** Occurrence of mental disorders comparing mastectomy, breast-conserving treatment (BCT), and patients with breast reconstruction.

Surgical Therapy	BCT 1	Mastectomy 2	Reconstruction 3	*p*-Value	Effect Size Cohens W	Posthoc Tests OR [95% CI]; Effect Size Cohens W (Significant Values Printed in Bold Letters)
N = 9365	N = 6188 (66.1%)	N = 2497 (26.7%)	N = 680 (7.3%)			
Mental disorder (total)	71.6% (N = 4428)	61.2% (N = 1529)	78.4% (N = 533)	**<0.01**	**0.112**	**1 vs. 2** **0.63** [0.57, 0.69], **0.10** 1 vs. 3 1.44 [1.19, 1.74], 0.05 **2 vs. 3** **2.30** [1.88, 2.80], **0.15**
Affective disorder	37.1% (N = 2296)	33.2% (N = 830)	45.3% (N = 308)	**<0.01**	0.061	1 vs. 2 0.84 [0.77, 0.93], 0.04 1 vs. 3 1.40 [1.20, 1.65], 0.05 **2 vs. 3** **1.66** [1.40, 1.98], **0.10**
Anxiety disorder	21.7% (N = 1345)	17.8% (N = 445)	26.6% (N = 181)	**<0.01**	0.057	1 vs. 2 0.78 [0.69, 0.88], 0.04 1 vs. 3 1.31 [1.09, 1.56], 0.04 2 vs. 3 1.67 [1.37, 2.04], 0.09
Obsessive compulsive disorder	0.2% (N = 14)	0.2% (N = 4)	0.4% (N = 3)	0.37	0.014	-
Dissociative disorder	0.6% (N = 39)	0.5% (N = 12)	1.2% (N = 8)	0.115	0.021	-
Hypochondriac disorder	40.8% (N = 2526)	30.9% (N = 772)	47.6% (N = 423)	**<0.01**	**0.103**	1 vs. 2 0.65 [0.59, 0.72], 0.09 1 vs. 3 1.32 [1.13, 1.55], 0.04 **2 vs. 3** **2.034** [1.71, 2.42], **0.14**
Other neuroses	12.2% (N = 754)	7.1% (N = 177)	16.8% (N = 114)	**<0.01**	0.086	1 vs. 2 0.55 [0.46, 0.65], 0.75 1 vs. 3 1.45 [1.17, 1.80], 0.04 **2 vs. 3** **2.64** [2.05, 3.40], **0.14**
Mania and bipolar disorder	0.3% (N = 17)	0.3% (N = 8)	0.4% (N = 3)	0.739630	0.008	-
Adjustment disorder	30.7% (N = 1902)	23.7% (N = 591)	43.1% (N = 293)	**<0.01**	**0.106**	1 vs. 2 0.70 [0.63, 0.78], 0.07 1 vs. 3 1.71 [1.45, 2.00], 0.08 **2 vs. 3** **2.44** [2.04, 2.92], **0.18**

Chi-squared tests (plus post hoc analyses), *p*-value < 0.01 considered significant given an effect size > 0.1 (Cohen’s W). Post hoc analyses: chi-squared tests *p*-value < 0.01 considered significant given an effect size > 0.1 (Cohen’s W), including the respective odds ratios (ORs) with a 95% confidence interval. Significant results are printed in bold.

## Data Availability

The data were provided by AOK Baden-Wuerttemberg. Due to privacy reasons and data security regulations, the data are only available with the consent of AOK Baden-Wuerttemberg.
